# Translator Exposure APIs: Open Access to Data on Airborne Pollutant Exposures, Roadway Exposures, and Socio-Environmental Exposures and Use Case Application

**DOI:** 10.3390/ijerph17145243

**Published:** 2020-07-21

**Authors:** Alejandro Valencia, Lisa Stillwell, Stephen Appold, Saravanan Arunachalam, Steven Cox, Hao Xu, Charles P. Schmitt, Shepherd H. Schurman, Stavros Garantziotis, William Xue, Stanley C. Ahalt, Karamarie Fecho

**Affiliations:** 1 Environmental Sciences and Engineering Department, University of North Carolina at Chapel Hill, Chapel Hill, NC 27599, USA; valenal@email.unc.edu; 2 Renaissance Computing Institute, University of North Carolina at Chapel Hill, Chapel Hill, NC 27517, USA; lisa@renci.org (L.S.); scox@renci.org (S.C.); xuhao@renci.org (H.X.); ahalt@renci.org (S.C.A.); 3 Kenan-Flagler Business School, University of North Carolina at Chapel Hill, Chapel Hill, NC 27599, USA; appolds@email.unc.edu; 4 Institute for the Environment, University of North Carolina at Chapel Hill, Chapel Hill, NC 27517, USA; sarav@email.unc.edu; 5 National Institute of Environmental Health Sciences, Durham, NC 27709, USA; charles.schmitt@nih.gov (C.P.S.); shepherd_schurman@nih.gov (S.H.S.); garantziotis@niehs.nih.gov (S.G.); william.xue@nih.gov (W.X.); 6 Copperline Professional Solutions, Pittsboro, NC 27312, USA

**Keywords:** airborne pollutants, roadway exposure, demographic factors, socio-economic factors, asthma, asthma exacerbations, open data, application programming interfaces, environmental health, public health

## Abstract

Environmental exposures have profound effects on health and disease. While public repositories exist for a variety of exposures data, these are generally difficult to access, navigate, and interpret. We describe the research, development, and application of three open application programming interfaces (APIs) that support access to usable, nationwide, exposures data from three public repositories: airborne pollutant estimates from the US Environmental Protection Agency; roadway data from the US Department of Transportation; and socio-environmental exposures from the US Census Bureau’s American Community Survey. Three open APIs were successfully developed, deployed, and tested using random latitude/longitude values and time periods as input parameters. After confirming the accuracy of the data, we used the APIs to extract exposures data on 2550 participants from a cohort within the Environmental Polymorphisms Registry (EPR) at the National Institute of Environmental Health Sciences, and we successfully linked the exposure estimates with participant-level data derived from the EPR. We then conducted an exploratory, proof-of-concept analysis of the integrated data for a subset of participants with self-reported asthma and largely replicated our prior findings on the impact of select exposures and demographic factors on asthma exacerbations. Together, the three open exposures APIs provide a valuable resource, with application across environmental and public health fields.

## 1. Introduction

Environmental exposures profoundly impact health and disease, both independently and by virtue of established (although not well understood) interactions with the genome [1]. A variety of publicly available data sources offer access to data on environmental exposures such as chemical hazards [2,3,4], water quality [5,6], air quality [7,8], and socio-environmental exposures [9,10,11]. However, even though these datasets are public, they are generally not easily obtained, navigated, or interpreted [12,13]. Moreover, many public environmental data sets are difficult to access and/or download in a form that is readily usable [14]. In addition, these data sets typically require field-specific knowledge and/or a specialized skill set that most public health researchers and environmental health scientists simply do not possess [15,16]. Compounding these challenges is an often lack of sufficient documentation to understand and interpret the data [17].

While the challenges presented by open environmental data have been well considered, less considered has been the need to move beyond epidemiological studies that demonstrate correlations in patterns of environmental exposures, such as airborne pollutant levels and population-based health outcomes. For example, many studies have demonstrated seasonal patterns in regional levels of pollen and correlation with emergency department visits for respiratory issues [18,19]. Other studies have identified associations between regional wildfires and increases in out-of-hospital cardiovascular events [20]. While such studies are quite powerful and provide insights into human health and disease, the ability to move beyond population-based studies in environmental health to individual-level studies allows for more mechanistic research on environmental exposures and health outcomes. One key capability to support such studies is the ability to openly access sufficiently granular exposures data and link the data to electronic health record data or research data such as survey responses in order to support analysis at the level of the individual, as opposed to the level of population.

The Biomedical Data Translator (“Translator”) program, funded by the National Center for Advancing Translational Sciences, aims to develop an open informatics platform to support the integration and semantic harmonization of disparate data sources and types and to enable interrogation of those data to inform a mechanistic characterization of health and disease [21,22,23]. As part of this work, we developed several open tools and services that provide users with easy access to processed and computable data, including the Integrated Clinical and Environmental Exposures Service (ICEES). ICEES is a Translator service that provides open access to electronic health record data that have been integrated at the individual level with a variety of public exposures data [24,25,26]. ICEES is currently focused on environmental influences on asthma-like conditions, but the service itself was developed as a disease-agnostic framework and approach for openly exposing integrated clinical and environmental exposures data.

Herein, we describe the research and development of three open application programming interfaces (APIs) that were motivated by our work on ICEES and a need to provide open access to individual-level environmental exposures data independent of ICEES, for application in environmental health research and epidemiological studies. The open availability of usable environmental exposures data will allow our team and others to identify mechanisms based on individual-level exposures and quantitatively assess their contribution to health outcomes. Indeed, the Translator architecture is based on distributed, open APIs to support nimble translational science, promote a culture of open team science, and facilitate translational science and collaboration beyond the Translator program [21,22,23]. Specifically, we describe: a Translator Airborne Pollutant Exposures API, which exposes nationwide data from the United States (US) Environmental Protection Agency (EPA); a Translator Roadway Exposures API, which exposes nationwide data from the US Department of Transportation; and a Translator Socio-environmental Exposures API, which exposes nationwide data derived from the US Census Bureau’s American Community Survey (ACS). We apply the data to a driving use case on a cohort within the Environmental Polymorphisms Registry (EPR) at the National Institute of Environmental Health Sciences (NIEHS).

## 2. Materials and Methods

### 2.1. Datasets

#### 2.1.1. Airborne Pollutant Exposures Data

Airborne pollutant estimates were derived from the US EPA’s data fusion method, namely, the Bayesian space-time “downscaler model” [27]. The data sets from this model provide 8-h ozone daily maxima and daily averages for particulate matter ≤ 2.5-µm in diameter (PM_2.5_). The data set sources fused by the downscaler originate from air quality monitoring data from the National Air Monitoring Stations and State or Local Air Monitoring Stations (NAMS/SLAMS) and numerical outputs from the Models-3/Community Multiscale Air Quality (CMAQ) modeling system [28]. The downscaler model calibrates CMAQ data using monitoring data and uses this relationship to estimate “observed” concentrations at new locations within a domain [29]. Ultimately, the estimates are returned at a resolution of US Census Tract centroids (*n* = 72,282) encompassed by the 12-km CMAQ modeling domain throughout the contiguous United States. Additional information on the downscaler method and its inputs can be found at the US EPA’s Remote Sensing Information Gateway [8]. Data from calendar years 2002 to 2016 (i.e., the most recent years available) were incorporated into the Translator Airborne Pollutant Exposures API.

#### 2.1.2. Roadway Exposures Data

Roadway data were collected from GIS shapefiles provided by the United States Federal Highway Administration (FHWA)’s Highway Performance Monitoring System (HPMS). The HPMS Field Manual contains a detailed explanation of methods and data fields [30]. Results are limited to major (or primary) road types, including interstates, principal arteries, minor arteries, and major collectors. For the Translator Roadway Exposures API, data from the 2016 calendar year were incorporated (i.e., the most recent year available). The data set contained data on 7.5 million major road segments and covered the contiguous United States. For the majority of road segments in the data set, information was available on road type, route identifier, maximum speed limit, total number of lanes, and annual average daily traffic (AADT) [31].

The HPMS data included values for road type, AADT, and speed limit for most, but not all, road segments. For HPMS road segments that did not contain road type, that information was obtained using TIGER (Topologically Integrated Geographic Encoding and Referencing) data from the US Census Bureau [32]. For HPMS road segments that were missing AADT and/or speed limit values, we employed a variety of approaches to assign those values. A lookup table was built with county averages by road type and populated with values from road segments that had values for AADT and/or speed limit. Most of the road segments were populated using this method. For certain road types (e.g., ramps) that lacked AADT and/or speed limit values, speed limits were based on the function class of the road and state averages for AADT. Any remaining road segments without a road type were removed from the dataset. Ultimately, ~5% of the road segments were dropped from the final data set exposed via the Translator Roadway Exposures API.

#### 2.1.3. Socio-Environmental Data

Socio-environmental data were extracted from two releases of ACS data for the US (50 states plus the District of Columbia), for the 2007–2011 and 2012–2016 survey periods. The ACS is the US Census Bureau’s largest household survey [33]. Approximately two million households have completed the survey annually since 2005. To preserve confidentiality, while maximizing geographic detail, the US Census Bureau releases summary data built from five annual waves at the US Census Block Group level (*n* = 217,739). Block Groups, the smallest geographic area for which ACS data are released, generally contain data on 600 to 3000 people. (These aggregate into Census Tracts, which have a target population size of 4000. Census Tracts aggregate into counties, and counties aggregate into states.) US Census Bureau geography, even Block Groups, may cross municipal lines and may include both urbanized and rural areas. Because population size is a large factor in determining US Census Bureau geography, some Block Groups may cover an extensive rural or wilderness area, while others are quite compact. Because the US Census Bureau is mandated to preserve the confidentiality of individual data, and because the variables of interest may not apply to all Block Groups, missing data can occur in a small number of situations [34]. In particular, calculated proportions were suppressed in Block Groups where the denominator (i.e., population, households, or population subset) was less than 50.

For the Translator Socio-environmental Exposures API, a range of variables relevant to environmental health research were extracted. Standard errors for the estimates were calculated whenever replicates were available. The variables that were extracted included: (1) population count; (2) residential density; (3) median household income; (4) proportion of the population who are not non-Hispanic white; (5) proportion of adults ≥ 25 years of age with a high school degree as highest maximum level of education; (6) proportion of households without an automobile; (7) proportion of the population without health insurance; (8) proportion of the population ≥ 5 years of age speaking a language other than English at home; (9) two alternative measures of the proportion of teenagers who are high school dropouts; (10) two alternative measures of the proportion of households receiving public assistance; (11) proportion of adult males aged 16–64 years who worked less than 26 weeks during the previous year; (12) proportion of households headed by a female (no spouse); (13) proportion of households headed by a female with a biological child; (14) proportion of households headed by a female with any child; (15) proportion of households receiving social security income; (16) proportion of households receiving at least partial governmental assistance; (17) proportion of adult males with little work; and (18) proportion of adults who are not employed. In addition, each Block Group was classified as being in a rural or urban area.

### 2.2. Application Programming Interface (API) Development

The Translator Exposures APIs were created using the Swagger OpenAPI platform. The Swagger toolset facilitates the automatic generation of an OpenAPI document (YAML format). The Swagger Codegen tool is used to create boilerplate source code for implementation of the API. The APIs described herein were created using the Python/Flask code generator. The Python/Flask API template was then fleshed out with code logic to process API input parameters and form appropriate SQL queries to return requested exposures data.

A PostgreSQL database with PostGIS extension was used for each API. The sqlacodegen tool was used to generate database schema models and class definitions that are used by the SQLAlchemy and GeoAlchemy Python packages. SQLAlchemy and GeoAlchemy were used in the API logic to perform database queries for calculating distances and other spatial relationships.

SQL, Python, and bash scripts were created to load CSV and shapefile data into the database. Most of the exposures data sets were quite large and required the development of custom Python applications that implemented high-performance data manipulation libraries in order to load the data in a timely manner.

The SQL databases for each API also required tuning to handle queries against large amounts of exposures data and calculate spatial relationships between data sets. Indexes were created for common query data relationships and geospatial proximity comparisons.

The APIs were tested initially using random input latitude and longitude values and default settings for other input parameters. After verifying the output, we applied the APIs to the use case described below.

### 2.3. Motivation and Application Use Case

#### 2.3.1. Study Population

Our application use case for research and development of the Translator Exposures APIs was a cohort of participants in the NIEHS EPR. The registry, which originated ~20 years ago and is comprised of ~20,000 study participants, is a resource dedicated to the advancement of the study of interactions between environmental and genetic determinants of health and disease. Study participants contributed survey-derived data on exposures and disease, in addition to biological samples (e.g., blood). We aimed to use the Translator Exposures APIs to extract participant-level data on airborne pollutant exposures, roadway exposures, and socio-environmental exposures and then integrate the data with EPR data on a cohort created to foster understanding of the role of inflammatory processes in asthma and other conditions [35]. For research and development of the Translator Exposures APIs, we focused on 2550 study participants who were successfully geocoded from a total of 4130 total participants in the EPR cohort on inflammation and respiratory conditions. For use case analysis and comparison with prior results [24,25,26,35,36], we focused on a subset of the cohort with a self-reported diagnosis of asthma (described below and in greater detail in the Results section, under Use Case Results and Table 2).

#### 2.3.2. Statistical Analysis

An exploratory analysis of the integrated data was conducted using summary statistics and Chi Square tests to examine the impact of select demographic and exposures variables. The variables that were selected were representative of each of the data sources (i.e., EPR data, Airborne Pollutant Exposures API, Roadway Exposures API, Socio-environmental Exposures API). Importantly, they were also chosen to replicate and extend our prior work [24,25,26,35,36], which focused on associations between asthma exacerbations and sex, race, history of smoking, obesity, airborne particulate matter exposure, major roadway or highway exposure, and estimated household income. We used those same variables for the demonstration project reported here. As noted above, we focused on a subset of the EPR cohort with a self-reported diagnosis of asthma (survey question: “Has a doctor or other health care provider ever told you that you have...asthma (Yes/No)?”) and a primary endpoint measure of self-reported emergency department (ED) or urgent care visits for asthma (survey question: “In the past 12 months, have you visited the ER or Urgent Care center because of asthma?”) as a proxy for asthma exacerbations in order to facilitate comparison with our prior work. We hypothesized that the Translator Exposures APIs could be used to replicate our prior findings and demonstrate that asthma exacerbations are more common among: females; African Americans; participants with a history of smoking; obese participants; participants exposed to relatively high levels of particulate matter; participants living within close proximity to major roadways or highways; and participants with relatively low estimated household income. The significance level was set at α = 0.10.

## 3. Results

### 3.1. User Interfaces (UIs)

**Figure 1:**
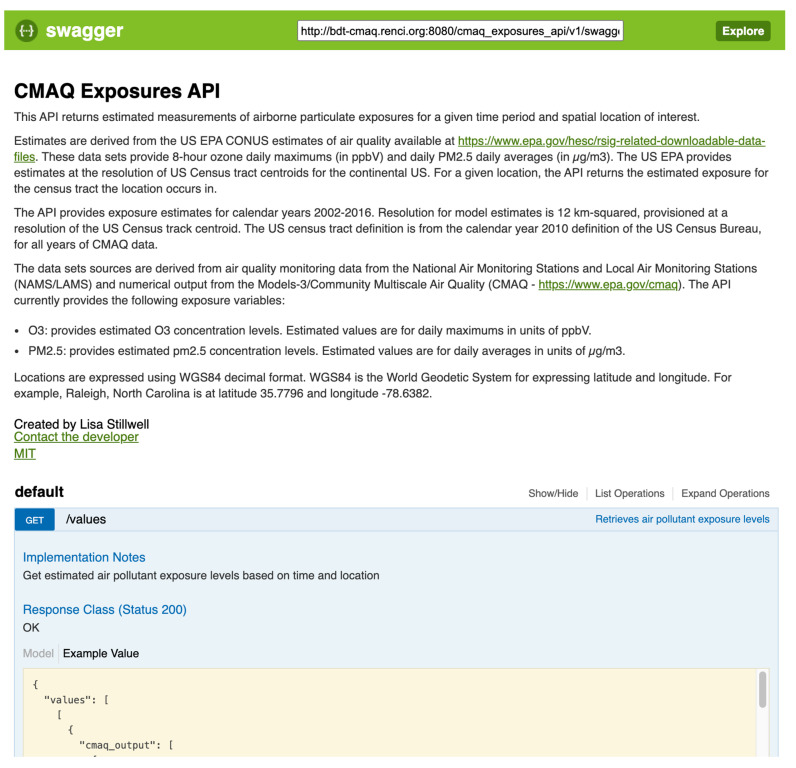
Screenshot of Swagger user interface to Translator Airborne Pollutant Exposures application programming interfaces (API).

Figure 1 provides a screenshot of the Swagger UI for the Translator Airborne Pollutant Exposures API, through which the environmental exposures data can be accessed. Similar interfaces exist for the Translator Roadway Exposures API and the Translator Socio-environmental Exposures API. Although the APIs can be accessed via command-line code, the Swagger UIs provide user-friendly interfaces, with hyperlinks to user documentation and sample queries, as well as contact information for the developer.

### 3.2. API Functionalities

The functionalities of the three Translator Exposures APIs are complementary. For each API, a latitude and longitude value are required input parameters. For the Airborne Pollutant Exposures API, users also must enter a start date and an end date, as well as a UTC time zone offset. For the Roadway Exposures API, a maximum distance (in meters) is required (500 m is the maximum allowable distance). For the Socio-environmental Exposures API, a survey period must be selected. In response to the input parameters, the services return the estimated environmental exposures for the indicated geospatial location and, when applicable, the time period of interest. The input file format is JSON, as is the output file format, and batch service requests are possible. Users may also enter input and receive output directly from the Swagger UIs.

**Figure 2:**
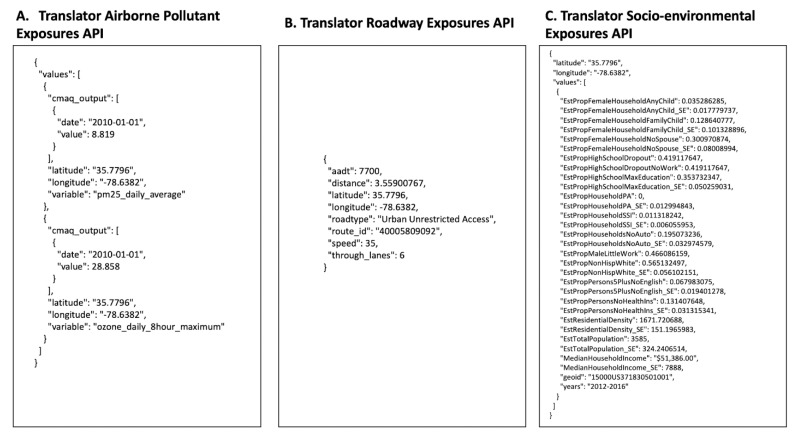
Example JSON output for Raleigh, North Carolina (latitude, longitude: 35.7796, −78.6382) for the Airborne Pollutant Exposures API (**A**), Roadway Exposures API (**B**), and Socio-environmental Exposures API (**C**). A start and an end date of 01–01–2010 and 01–01–2010 was used for the Airborne Pollutant Exposures API; a default setting of 500 m was used for the Roadway Exposures API; the 2012–2016 survey period was selected for the Socio-environmental Exposures API; and the UTC time zone offset was chosen.

For all three APIs, results are provided for all available data types for a given API. Figure 2 shows example JSON output for each API, using the latitude and longitude of (35.7796, −78.6382 [Raleigh, North Carolina]) as input parameters for all three APIs, a start and an end date of (01–01–2010, 01–01–2010) for the Airborne Pollutant Exposures API, a survey period of (2012–2016) as the study period for the Socio-environmental Exposures API, and default settings of 500 m for the Roadway Exposure API, with UTC for the time zone offset.

### 3.3. API Data Extraction

We successfully used the three Translator Exposures APIs to extract data on 2550 study participants who were successfully geocoded from a total of 4130 total participants in the overall EPR cohort on inflammation and respiratory conditions (Table 1). The input parameters consisted of the geocode (latitude/longitude) for participant self-reported primary residence as the spatial location of interest and the calendar year 2014 as the time period of interest. Of note, we chose calendar year 2014 because that was the year that the majority of participants in the cohort completed the survey. Default settings were used for other input parameters. One hundred percent of available data fields was successfully extracted from each of the three APIs, by direct batch request to each endpoint. Importantly, after successfully extracting the exposures data, we were able to link the data to participant-level EPR data on all 2550 participants who were successfully geocoded.

### 3.4. Use Case Results

To facilitate comparison with prior EPR findings on participants with self-reported asthma [35] and results from ICEES on patients with asthma-like conditions as documented in electronic health records [24,25,26,36], we focused our preliminary analysis on an EPR subcohort of *n* = 932 participants with a self-reported diagnosis of asthma, of which *n* = 923 participants had data on the primary endpoint measure of self-reported ED or urgent care visits for asthma (one or more in prior 12 months), and *n* = 879 of those had geocodes and therefore had exposures data available from the Translator Exposures APIs. We explored the impact of select demographic features and environmental exposures on asthma exacerbations, chosen again to facilitate comparison with our prior work: sex (from EPR); race (from EPR); history of smoking (from EPR); obesity (from EPR); exposure to PM_2.5_ (from Airborne Pollutant Exposures API) exposure to a major roadway or highway (from Roadway Exposures API); and estimated median household income exposure (from Socio-environmental Exposures API). Self-reported ED or urgent care visits for asthma (one or more in prior 12 months) (from EPR) were used as a proxy for asthma exacerbations and served as the primary endpoint measure.

We found that the proportion of participants reporting ED or urgent care visits for asthma was higher among females versus males (*p* < 0.10), African Americans versus non–African Americans (*p* < 0.0001), participants with a history of smoking versus those without a history of smoking (*p* < 0.10), obese participants versus non-obese participants (*p* < 0.001), and participants with low median household income versus those with higher household income (*p* < 0.001) (Table 2). A similar, albeit non-significant, association was found for exposure to PM_2.5_, with the proportion of participants reporting ED or urgent care visits for asthma increasing with exposure to increasing levels of PM_2.5_ (*p* = 0.13). No relationship was found between participant self-report of ED or urgent care visits for asthma and exposure to major roadways or highways.

## 4. Discussion

We developed three open Translator Exposures APIs in response to a need to openly expose usable, individual-level data on environmental exposures that are known to impact health and disease, and in an effort to facilitate research by our team and others in environmental health, epidemiology, and related fields. We successfully used the open APIs to extract exposures data on 100% of geocoded participants within an EPR cohort, and we integrated the exposures data with EPR data at the participant level. Importantly, we applied the data to a proof-of-concept asthma use case and demonstrated an association between asthma exacerbations, as measured by participant self-report of ED or urgent care visit for asthma, and sex, race, smoking history, obesity, median household income, and exposure to airborne particulate matter, thus largely supporting our hypothesis that the Translator Exposures APIs could be used to replicate our prior findings [24,25,26,35,36].

While we were able to largely replicate our prior findings, certain key differences in both the methodological approach and the findings should be noted. First, we adopted a primary endpoint of self-reported ED or urgent care visits for asthma in the present study, whereas in our prior EPR study [35], we were more inclusive and adopted a primary endpoint that included self-reported ED or urgent care visits for asthma plus activity limitations and sleeplessness (examined individually and collectively). We intentionally focused only on self-reported ED visits for asthma in order to facilitate comparison with both the prior EPR study and our prior findings on ICEES [24,25,26,36], which invoked—as a primary endpoint—ED or inpatient visits for respiratory issues, as documented in electronic health records. Another difference between the prior EPR study and the present one is that we focused the present work on a subset of EPR participants who had a self-reported diagnosis of asthma, whereas our prior EPR study included participants with other conditions. As with our choice of primary endpoint, the decision to focus on a subset of the broader cohort was again intended to facilitate comparison with both our prior EPR study and our prior ICEES findings.

Another difference between the present work and our prior work is that the association between asthma exacerbations and exposure to airborne particulate matter was not significant in the use case reported here, in contrast to our findings with ICEES [24,25,26,36] and despite the fact that exposure to airborne pollutants is a well-established risk factor for asthma [37,38,39,40]. Nonetheless, a clear linear trend was apparent in the present work, with increases in exposure levels associated with increases in participant self-report of ED or urgent care visits for asthma.

We also did not identify an association between exposure to major roadways or highways and asthma exacerbations in participants with self-reported asthma, in contrast to our prior EPR study [35], in which we identified an association between exposure to major roadways or highways and asthma-related morbidity in a subset of survey participants with genetically defined susceptibility to airborne pollutants. Other groups have likewise linked traffic flow and traffic-related airborne pollutants to adverse respiratory events (e.g., [41]). We plan to conduct a more rigorous multivariate analysis to explore interactions between the features reported here and others, including single nucleotide polymorphisms. We also plan to refine the roadway metric to better account for the fact that the study population is largely rural, with only ~20% of participants residing within 100 m of a major roadway or highway. We emphasize that because the API returns an estimated distance for any given input latitude and longitude, the limitation of our roadway metric is a reflection of our application use case, not the API.

The present study, our prior studies [24,25,26,35,36], and those of other groups [40,41,42,43,44,45,46,47,48] consistently identified associations between obesity, minority race, low socio-economic status, and asthma or asthma-related morbidity. For example, communities of low socioeconomic status face higher concentrations of airborne pollutants [42,43], and studies have demonstrated that non-whites are disparately exposed to traffic and air pollution [44,45]. Moreover, minority race and low socio-economic status have been associated with asthma and asthma-related morbidity [26,35,46,47,48], perhaps by virtue of geographical location and exposure to high levels of air pollution. In addition, we and many others have demonstrated a relationship between obesity or history of smoking and increased risk of asthma exacerbations [35,36], as was also shown here. The relationship between sex and asthma is less consistent in the published literature, although as with the current study, our prior EPR study [35] likewise found that females were more likely than males to self-report various survey measures of asthma-related morbidity.

## 5. Conclusions

The findings reported here demonstrate the utility of the open Translator Exposures APIs and their application in a proof-of-concept use case, which largely supported our hypothesis. We plan to continue research and development of these open services and expand the available data and functionalities. For instance, we recently acquired and processed data on additional airborne pollutants (i.e., acetaldehyde, formaldehyde, CO, NO, NO_2_, NO_x_, SO_2_, and benzene), and we plan to make those data available via the Airborne Pollutant Exposures API. In addition, we plan to offer an option to conduct statistical calculations on the data (e.g., cumulative airborne pollutant exposures) in order to better model exposure history. We also are exploring the possibility of creating an integrated Translator Exposures API that would expose the underlying integrated data via an interactive, spatiotemporal, nationwide visual map, in order to allow users to explore exposure clusters across the continental United States. In addition, we are expanding the demonstration use case on asthma to include additional exposure variables and more rigorous statistical analyses (e.g., generalized linear methods, regression models). Finally, we are developing new use cases and identifying new public exposures data to further demonstrate the utility of the open Translator Exposures APIs and increase our user base. In conclusion, we anticipate that the Translator Exposures APIs will have broad applicability across many fields within environmental research and public health.

## Figures and Tables

**Table 1:** EPR data extraction and integration results for the three open Translator Exposures APIs.

General Statistics	Comments
4130 total participants	all participants had a self-reported diagnosis of asthma
2550 participants with geocodes	5 participants had a foreign address; one participant resided in Alaska/Hawaii; 1576 participants were not geocoded due to home addresses that were listed as P.O. boxes or routes, or addresses that were outdated, incomplete, or missing
2550 participants with complete airborne pollutant exposures data	extraction included the number of observations per airborne pollutant per participant
2550 participants with complete roadway data	954 participants (37.4%) had a primary residence located 500 m or greater from a major roadway (i.e., the maximum distance reported by the API)
2550 participants with complete ACS data	extraction included standard error estimates for each socio-environmental exposure, as those are available via the API as standard ACS metrics
2550 participants with integrated exposures and survey data	integration was at the participant level

Note: ACS = American Community Survey; EPR = Environmental Polymorphisms Registry.

**Table 2:** EPR data on a subset of participants with a self-report of asthma, integrated at the participant-level with data derived from the Translator Exposures APIs.

Feature Variable	0 ED or Urgent Care Visits for Asthma Prior 12 Months	1+ ED or Urgent Care Visits for Asthma Prior 12 Months	TOTAL *n* (Row %) (Column %)	*p* Value ^†^
	n * (Row %) (Column %)	*n* (Row %) (Column %)		
	*n* = 806	*n* = 117	*n* = 923	
**Sex**				*p* < 0.10
Female	593 (86.2%) (73.6%)	95 (13.8%) (81.2%)	688 (100.0%) (74.5%)	
Male	213 (90.6%) (26.4%)	22 (9.36%) (18.8%)	235 (100.0%) (25.5%)	
**Race**				*p* < 0.0001
African American	179 (77.5%) (22.2%)	52 (22.5%) (47.3%)	231 (100.0%) (25.0%)	
Caucasian	562 (90.6%) (69.7%)	58 (9.35%) (52.7%)	620 (100.0%) (67.2%)	
Other	65 (90.3%) (8.06%)	7 (9.72%) (6.36%)	72 (100.0%) (7.80%)	
	*n =* 799	*n =* 117	*n =* 916	
**History of Smoking**				*p* < 0.10
Yes	327 (84.7%) (40.9%)	59 (15.3%) (50.4%)	386 (100.0%) (42.1%)	
No	472 (89.1%) (59.1%)	58 (10.9%) (49.6%)	530 (100.0%) (57.9%)	
	*n =* 790	*n =* 114	*n =* 904	
**Obese**				*p* < 0.0001
Yes	345 (85.0%) (43.7%)	61 (15.0%) (53.5%)	406 (100.0%) (44.9%)	
No	445 (89.4%) (56.3%)	53 (10.6%) (46.5%)	498 (100.0%) (55.1%)	
	*n =* 773	*n =* 106	*n =* 879	
**Average Annual PM_2.5_ Exposure ^‡^**				*p* = 0.13
Bin 1 (4.64, 7.99] μg/m^3^	20 (100.0%) (2.59%)	0 (0.00%) (0%)	20 (100.0%) (2.28%)	
Bin 2 (7.99, 9.66] μg/m^3^	149 (90.3%) (19.3%)	16 (9.70%) (15.1%)	165 (100.0%) (18.8%)	
Bin 3 (9.66, 13.01] μg/m^3^	604 (87.0%) (78.1%)	90 (13.0%) (84.9%)	694 (100.0%) (79.0%)	
**Major Roadway Exposure**				*p* = 1.00
≤250 m	332 (87.6%) (42.9%)	47 (12.4%) (44.3%)	379 (100.0%) (43.1%)	
>250 m	441 (88.2%) (57.1%)	59 (11.8%) (55.7%)	500 (100.0%) (56.9%)	
**Median Household Income Exposure ^‡^**				*p* < 0.001
Bin 1 (7538, 48,694] US$	311 (83.2%) (40.2%)	63 (16.8%) (59.4%)	374 (100.0%) (42.5%)	
Bin 2 (48,694, 89,646] US$	355 (90.6%) (45.9%)	37 (9.44%) (34.9%)	392 (100.0%) (44.6%)	
Bin 3 (89,646, 212,500] US$	107 (94.7%) (13.8%)	6 (5.31%) (5.66%)	113 (100.0%) (12.9%)	

Abbreviations: ED, emergency department; PM_2.5_, particulate matter ≤ 2.5-µm in diameter. * Sample sizes reflect the starting sample size of *n* = 932 EPR participants with a self-reported diagnosis of asthma, of which *n* = 923 participants had data on the primary endpoint of self-reported ED or urgent care visits for asthma (one or more in prior 12 months), and *n* = 879 of those had geocodes and therefore had exposures data available from the Translator Exposures APIs. Sample sizes vary by feature variable due to missing data arising from incomplete surveys. ^†^ Significance level was set at *p* < 0.10. ^‡^ Binned using pandas cut function to facilitate comparison with [24] and account for the granularity of the data, with airborne pollutant exposure estimates obtained at a resolution of 12 km-squared and provisioned at a resolution of the US Census Track centroid, and median household income estimates provisioned at the US Census Block Group level.

## Data Availability

Data from the Translator Exposures APIs are openly accessible at: http://bdt-cmaq.renci.org:8080/cmaq_exposures_api/v1/ui/; http://bdt-proximity.renci.org:8080/roadway_proximity_api/v1/ui/; http://bdt-social.renci.org:8080/socio_environmental_exposures_api/v1/ui/.

## Software Availability

GitHub repositories for the Translator Exposures APIs are openly accessible at:

https://github.com/lstillwe/cmaq-exposures-api;

https://github.com/lstillwe/roadway-proximity-api;

https://github.com/lstillwe/socio-economic-api.

## References

[1] Snyder-Mackler N., Sanz J., Kohn J.N., Voyles T., Pique-Regi R., Wilson M.E., Barreiro L.B., Tung J. (2019). Social status alters chromatin accessibility and the gene regulatory response to glucocorticoid stimulation in rhesus macaques. Proc. Natl. Acad. Sci. USA.

[2] Dionisio K.L., Phillips K., Price P.S., Grulke C.M., Williams A., Biryol D., Hong T., Isaacs K.K. (2018). The Chemical and Products Database, a resource for exposure-relevant data on chemicals in consumer products. Sci. Data.

[3] US EPA Chemical and Products Database (CPDat). http://www.epa.gov/chemical-research/chemical-and-products-database-cpdat.

[4] US EPA ACToR. http://actor.epa.gov/actor/home.xhtml.

[5] US EPA Drinking Water Treatability Database (TDB). http://www.epa.gov/water-research/drinking-water-treatability-database-tdb.

[6] US EPA Ecotoxicology Database. http://www.epa.gov/chemical-research/ecotoxicology-database.

[7] US EPA Air Quality System (AQS). http://www.epa.gov/aqs.

[8] US EPA RSIG-Related Downloadable Data Files. http://www.epa.gov/hesc/rsig-related-downloadable-data-files.

[9] US EPA Consolidated Human Activity Database (CHAD). http://www.epa.gov/healthresearch/consolidated-human-activity-database-chad-use-human-exposure-and-health-studies-and.

[10] CDC National Environmental Public Health Tracking Network. http://ephtracking.cdc.gov/.

[11] CDC CDC WONDER. http://wonder.cdc.gov/.

[12] Dvir N. (2018). Mitigating Challenges of Open Government Data.

[13] Roberts T. (2012). The Problem with Open Data. http://www.computerweekly.com/opinion/The-problem-with-Open-Data.

[14] Martin C. (2014). Barriers to the open government data agenda: Taking a multi-level perspective. Policy Internet.

[15] Martin S., Foulonneau M., Turki S., Ihadjadene M., Paris U., Tudor P. (2013). Risk analysis to overcome barriers to open data. Electron. Gov..

[16] Dawes S.S., Vidiasova L., Parkhimovich O. (2016). Planning and designing open government data programs: An ecosystem approach. Gov. Inf. Q..

[17] Gascó-Hernández M., Martin E.G., Reggi L., Pyo S., Luna-Reyes L.F. (2018). Promoting the use of open government data: Cases of training and engagement. Gov. Inf. Q..

[18] Silverman R.A., Stevenson L., Hastings H.M. (2003). Age-related seasonal patterns of emergency department visits for acute asthma in an urban environment. Ann. Emerg. Med..

[19] Wisniewski J.A., McLaughlin A.P., Stenger P.J., Patrie J., Brown M.A., El-Dahr J.M., Platts-Mills T.A., Byrd N.J., Heymann P.W. (2016). A comparison of seasonal trends in asthma exacerbations among children from geographic regions with different climates. Allergy Asthma Proc..

[20] Haikerwal A., Akram M., Del Monaco A., Smith K., Sim M.R., Meyer M., Tonkin A.M., Abramson M.J., Dennekamp M. (2015). Impact of fine particulate matter (PM2.5) exposure during wildfires on cardiovascular health outcomes. J. Am. Heart Assoc..

[21] Austin C.P., Colvis C.M., Southall N.T. (2019). Deconstructing the translational tower of babel. Clin. Transl. Sci..

[22] The Biomedical Data Translator Consortium (2019). The Biomedical Data Translator program: Conception, culture, and community. Clin. Transl. Sci..

[23] The Biomedical Data Translator Consortium (2019). Toward a universal biomedical data translator. Clin. Transl. Sci..

[24] Fecho K., Pfaff E., Xu H., Champion J., Cox S., Stillwell L., Peden D.B., Bizon C., Krishnamurthy A., Tropsha A. (2019). A novel approach for exposing and sharing clinical data: The Translator Integrated Clinical and Environmental Exposures Service. J. Am. Med. Inform. Assoc..

[25] Pfaff E.R., Champion J., Bradford R.L., Clark M., Xu H., Fecho K., Krishnamurthy A., Cox S., Chute C.G., Overby Taylor C. (2019). Fast Healthcare Interoperability Resources (FHIR) as a meta model to integrate common data models: Development of a tool and quantitative validation study. JMIR Med. Inform..

[26] Xu H., Cox S., Stillwell L., Pfaff E., Champion J., Ahalt S.C., Fecho K. (2020). FHIR PIT: An open software application for spatiotemporal integration of clinical data and environmental exposures data. BMC Med. Inform. Decis. Mak..

[27] Berrocal V.J., Gelfand A.E., Holland D.M. (2010). A spatio-temporal downscaler for output from numerical models. J. Agric. Biol. Environ. Stat..

[28] Byun D., Schere K.L. (2006). Review of the governing equations, computational algorithms, and other components of the Models-3 Community Multiscale Air Quality (CMAQ) Modeling System. Appl. Mech. Rev..

[29] US EPA (2018). Bayesian Space-Time Downscaling Fusion Model (Downscaler)—Derived Estimates of Air Quality for 2009.

[30] Federal Highway Administration (2018). Highway Performance Monitoring System (HPMS) Field Manual—Policy. http://www.fhwa.dot.gov/policyinformation/hpms/fieldmanual/page00.cfm.

[31] Federal Highway Administration (2016). AADT/ADT. http://www.fhwa.dot.gov/policyinformation/travel_monitoring/pubs/aadt/.

[32] US Census Bureau (2018). TIGER/Line® Shapefiles and TIGER/Line® Files. http://www.census.gov/geo/maps-data/data/tiger-line.html.

[33] US Census Bureau (2018). American Community Survey Data Profiles. http://www.census.gov/acs/www/data/data-tables-and-tools/data-profiles/2016/.

[34] US Census Bureau (2014). American Community Survey Design and Methodology.

[35] Schurman S.H., Bravo M.A., Innes C.L., Jackson W.B., McGrath J.A., Miranda M.L., Garantziotis S. (2018). Toll-like receptor 4 pathway polymorphisms interact with pollution to influence asthma diagnosis and severity. Sci. Rep..

[36] Ahalt S.C., Chute C.G., Fecho K., Glusman G., Hadlock J., Solbrig H., Overby Taylor C., Pfaff E., Ta C., Tatonetti N. (2019). Clinical data: Sources and types, regulatory constraints, applications. Clin. Transl. Sci..

[37] Delfino R.J., Coate B.D., Zeiger R.S., Seltzer J.M., Street D.H., Koutrakis P. (1996). Daily asthma severity in relation to personal ozone exposure and outdoor fungal spores. Am. J. Respir. Crit. Care Med..

[38] Mirabelli M.C., Vaidyanathan A., Flanders W.D., Qin X., Garbe P. (2016). Outdoor PM2.5, ambient air temperature, and asthma symptoms in the past 14 days among adults with active asthma. Environ. Health Perspect..

[39] Schildcrout J.S., Sheppard L., Lumley T., Slaughter J.C., Koenig J.Q., Shapiro G.G. (2006). Ambient air pollution and asthma exacerbations in children: An eight-city analysis. Am. J. Epidemiol..

[40] Urman R., McConnell R., Islam T., Avol E.L., Lurmann F.W., Vora H., Linn W.S., Rappaport E.B., Gilliland F.D., Gauderman W.J. (2014). Associations of children’s lung function with ambient air pollution: Joint effects of regional and near-roadway pollutants. Thorax.

[41] English P., Neutra R., Scalf R., Sullivan M., Waller L., Zhu L. (1999). Examining associations between childhood asthma and traffic flow using a geographic information system. Environ. Health Perspect..

[42] Pratt G.C., Vadali M.L., Kvale D.L., Ellickson K.M. (2015). Traffic, air pollution, minority and socio-economic status: Addressing inequities in exposure and risk. Int. J. Environ. Res. Public Health.

[43] Tian N., Xue J., Barzyk T.M. (2013). Evaluating socioeconomic and racial differences in traffic-related metrics in the United States using a GIS approach. J. Expo. Sci. Environ. Epidemiol..

[44] Marshall J.D., Swor K.R., Nguyen N.P. (2014). Prioritizing environmental justice and equality: Diesel emissions in Southern California. Environ. Sci. Technol..

[45] Rowangould G.M. (2013). A census of the US near-roadway population: Public health and environmental justice considerations. Transp. Res. Part D Transp. Environ..

[46] Alexander D., Currie J. (2017). Is it who you are or where you live? Residential segregation and racial gaps in childhood asthma. J. Health Econ..

[47] Keet C.A., Matsui E.C., McCormack M.C., Peng R.D. (2017). Urban residence, neighborhood poverty, race/ethnicity, and asthma morbidity among children on Medicaid. J. Allergy Clin. Immunol..

[48] Litonjua A.A., Carey V.J., Weiss S.T., Gold D.R. (1999). Race, socioeconomic factors, and area of residence are associated with asthma prevalence. Pediatr. Pulmonol..

